# New Orleans School of Medicine

**Published:** 1856-07

**Authors:** 


### NEW ORLEANS SCHOOL OF MEDICINE.

We have recently received the first annual announcement of this new school, and must say that we are highly pleased with some features of the new organization. With Dr. Erasmus and Dr. Fenner at its head, we bespeak for the school a brilliant future.

